# Simulation Exercises To Strengthen Polio Outbreak Preparedness in The Horn of Africa: Experiences and Lessons Learnt

**DOI:** 10.29245/2578-3009/2021/S2.1107

**Published:** 2021-04-05

**Authors:** Samuel Okiror, Chidiadi Nwogu, Obianuju Igweonu, Rustam Hydarov, Djiboui Karim, Farkhard Imambakiev, John Ogange, Annet Kisakye, Joseph Okeibunor, Hemant Shukla

**Affiliations:** 1 WHO Horn of Africa Coordination Office (HOA), Nairobi KENYA; 2 University of Nigeria, Nsukka; 3 UNICEF, Nairobi; 4 WHO headquarters, Geneva; 5 UNICEF New York; 6 World Health Organization, Kenya Country Office; 7 WHO, Uganda; 8 WHO Regional Office for Africa (WHO AFRO) Brazzaville, Congo; 9 WHO Headquarters, Geneva

**Keywords:** Poliovirus, Polio outbreak simulation exercise (POSE), Preparedness exercise, Polio eradication, HoA

## Abstract

**Background:**

Poliovirus importations and related outbreaks occurred in the Horn of Africa (HoA) following an initial outbreak, which started in Somalia, spread into Kenya within ten days and later into Ethiopia and gradually to other countries in the region. National preparedness plans for responding to poliovirus introduction were insufficient in many countries of the Region. We describe a series of polio outbreak simulation exercises that were implemented to formally test polio outbreak preparedness plans in the HoA countries, as a step to interrupting further transmission.

**Methods:**

The Polio Outbreak Simulation Exercises (POSEs) were designed and implemented. The results were evaluated and recommendations made. The roles of outbreak simulation exercises in maintaining regional polio-free status were assessed. In addition, we performed a comprehensive review of the national plans of all for seven countries in the HoA Region.

**Results:**

Seven simulation exercises, delivered between 2016 and 2017 revealed that participating countries were generally prepared for poliovirus introduction, but the level of preparedness needed improvement. The areas in particular need of strengthening were national preparedness plans, initial response, plans for securing vaccine supply, and communications.

**Conclusions:**

Polio outbreak simulation exercises can be valuable tools to help maintain polio-free status and should be extended to other high-risk countries and subnational areas in the HoA Region and elsewhere. There is also need to standardize the process and methods for conducting POSE for comparability.

## Introduction

The Global Polio Eradication Initiative (GPEI) has recorded tremendous progress in the fight to eradicate polio and make the world polio free^1–5^. However, the threat of outbreak of poliovirus (WPV) remains palpable in many places. Three countries, namely Afghanistan, Nigeria, and Pakistan, all within the African and Eastern Mediterranean Regions have remained endemic and importation- related outbreaks continue to occur in polio-free areas^2^ within the two Regions that constitute the Horn of Africa. The detection of new cases of wild polio virus (WPV) in the second half of 2016 was not only a setback but devastating for a region that was close to being certified polio free^1,6^. Thus the threat of polio will only be considered over when polio is completely eradicated when all regions are free of the risk for importation^7^.

Earlier between 2013 and 2014 there were series of outbreaks of wild poliovirus type 1 (WPV1) in the Horn of Africa. Six cases were confirmed, four from Somalia (Banadir and Bay region) and two from Kenya (Dadaab in north--eastern Kenya) in 2013. And as of 1 Jul 2013, 25 cases were reported from Somalia (primarily from Banadir region) and six from Kenya (Dadaab in north-eastern Kenya). The cases continued to rise as the year progressed. As of the end of 2013, the polio outbreak in the Horn of Africa was on the decline and the total number of polio cases stood at 203 (183 from Somalia, 14 from Kenya and six from Ethiopia). The most recent case in the region had onset of paralysis on 9 Oct 2014 (from Lower Shabelle, Somalia). By 18 Jun 2014, the total number of cases in the region was 219 since the beginning of the outbreak in Apr 2013 (195 from Somalia, 14 from Kenya and 10 from Ethiopia)^8^. By mid-2014, the outbreaks in the Horn of Africa that spanned 2013 and the first half of 2014 were brought to the verge of being stopped. Though the sporadic outbreaks of polioviruses in the Horn of Africa had been brought under control, risk assessments continued to show possibilities of outbreaks, given the performance of the region on surveillance indicators as well as routine immunization coverage in the area. This reality prompted the Horn of Africa Technical Advisory Group (TAG) to call for intensification of efforts in order to keep the virus permanently out of the region and complete the job^9^.

During its meeting in August 2014, the Horn of Africa TAG noted that national plans of action for responding to poliovirus circulation were not consistent with the Global Polio Eradication Initiative (GPEI) standard operating procedures and recommended conducting a formal test of the national preparedness plan in one or more appropriate countries. This recommendation led to the series of tabletop polio outbreak simulation exercises (POSEs) to explore national planning and coordination in response to detection of poliovirus circulation. The first four sets of POSE were conducted in Sudan, Ethiopia, Eritrea and Tanzania in 2016. Another three sets of POSEs were conducted in Kenya, South Sudan, and Uganda in 2017.

The exercises were designed to stimulate the countries to critically review and bring their national plans to be consistent with the standard operating procedures to responding to detection of polioviruses, to increase preparedness. The exercises addressed aspects of response, such as coordination, communication, and collaboration at international and national levels^2^. The objectives of the exercises included assessing preparedness for a possible event of WPV importation or cVDPV circulation, identifying preparedness strengths and challenges in individual countries, strengthening capacity to respond rapidly to poliovirus detection, improving country response and use of the International Health Regulations (IHR) mechanism in case of WPV detection, and exploring the communications response, including strengthening communications planning, use of social media tools, and management of traditional media outlets. This paper reviews and summarizes experiences garnered during the seven POSEs in the Horn of Africa between 2016 and 2017 with a focus on the country readiness to respond to a polio outbreak in line with the existing plan should one occur.

## Methods

The POSE series of tabletop exercises was designed and delivered by a core team made of the Horn of Africa Coordinating office in Kenya and WHO Headquarters as well as UNICEF ESARO for communication. The lead facilitators trained a team of evaluators drawn from various WHO country offices with considerable experience of implementation of polio eradication activities at national and sub-national level including familiarity with a wide range of knowledge on exercises to test preparedness of the country and its partners to responding to outbreaks of polio. Each POSE was country specific and engaged external facilitators. Participants received exercisespecific scenarios and acted upon this information to simulate realistic response activities. The exercises encouraged interaction and communication between groups. Participants had access to the Internet, plans, and information sheets.

Each POSE started with a presentation by the National team giving an overview of their National polio outbreak preparedness and response plan. This was followed by brief presentations by the Lead External Facilitators highlighting rules of the exercise. All information received through the different case scenarios should be considered as real life situations and each participant present had to know their roles and responsibilities in the polio preparedness and response activities. The team members were reminded to undertake the simulation exercise within the context of their National Response plan and use the findings and their experience to review and update the plan. External Facilitators made presentation of the new updates on SOPs. Brief discussion sessions on these presentations followed to clarify any unclear areas.

After the presentations and the discussions, the simulation exercise started with notification of the case. The National Task Force (NTF) through the Chair was provided with the different progressive case scenarios to respond to, which progressed over time to reflect the process of what could happen in response actions to control the VDPV outbreak and within the same setting. The simulation exercise took almost eight hours. Participants were cautioned by the facilitators not to fight against the scenario to what they liked rather than take them objectively and seriously. Two External Facilitators from WHO- Horn of Africa and UNICEF ESARO whose major role was to coordinate and manage the entire simulation exercise facilitated the simulation exercise. The facilitators specifically ensured the smooth running of the simulation by organizing and assisting participants in achieving the stated objectives of the simulation. They also provided guidance as deemed necessary as well as ensuring that all group members were engaged and participating. During the plenary discussions, the facilitators supported the NTF chair to ensure that the participants responded to the questions and issues raised. In regards with their overall observations of each phase of the scenarios, the facilitators then provided feedback. The delivered feedbacks by the facilitators at the end of each plenary session were very significant in updating the evaluations as well.

Evaluators were provided with an integrated checklist comprising of each scenario to enable them to document whether the input and expected actions would produce the desired outcome of the POSE. The evaluators answered three specific questions as follows; whether the aims and objectives of the exercise were achieved. What were the results? In addition, what improvement plans or performances are required in order to achieve the exercise aims and objectives? The evaluators assessed progressive scenario along with their feedback using the evaluation checklist. Table 1 provides a summary of the components to be evaluated, the scores range and a comments section to explain why a particular score was allocated to the component. A weighed score of each question by all the Evaluators was obtained and this was what was considered as the final score for that particular number, these were consolidated using excel sheet. The weighting of the score was based on identifying specific point or actions that should be included in the activities conducted and if any of those identified points or action missed there will be deductions in the scores according the total number of points generated in the answer for every question (the 4 degree are divided among them so there will be a specific value for each point). At the end of the exercise all participants on an individual basis were tasked to evaluate the exercise using a one page questionnaire and through their observations of each phase of scenario. They came up with strengths, gaps and suggestions/opportunities that they identified in the polio preparedness and response plan including establishing key bottlenecks in the implementation process of the response plan and ways of improving the plan.

Polio outbreak response simulation exercise (POSE) was conducted in a specific hypothetical outbreak area. The exercise comprised of seven progressive scenarios that mirrored what could happen during a real-life response in any country. The scenarios portrayed unfolding events to a polio outbreak response of approximately over a period of 120 days when the cVDPV outbreak should be interrupted once it occurs. The developed scenarios were sequenced beginning with; an initial outbreak notification and confirmation, followed by outbreak investigation, then rapid vaccination response, and finally to a crisis management of handling another reported case that developed in a different part of the country which necessitated a further mobilization of resources and refining of the plan. Altogether, the simulation exercise led to critical review of issues needed to be addressed to improve the national outbreak response plan and activities that would be undertaken once a Polio Virus is detected in the country.

Participants were given a set of seven scenarios and test exercises (See sample scenario in Table 2). Detailed list of scenarios are contained in Box 1. The same scenarios were used for all the countries. However, country specific issues were also raised in addition. As the simulated outbreaks developed, sessions covered local, national, and international communications; supplementary immunization activities (SIAs); enhanced surveillance; and local and reference laboratory activities. Participants were encouraged in each exercise to consider budgetary implications of their response plans. Discussions and feedback after each session were guided by the facilitators. Feedback from facilitators, observers, as well as participants’ responses, and debrief sessions, informed evaluation of each response plan.

### Evaluation of the polio outbreak simulation exercise

The external evaluators had a task of assessing responses to all scenario simulation activities. In each scenario the evaluators moved round to look at how activities of the various teams were being carried out. They made observations and analyzed all the scenarios by keenly looking and listening into how the activities complied with the recommended ‘National polio outbreak response plan’ and how active each team was playing their respective roles. They also assessed the presentations made in the plenary and took into account the comments of the external facilitators and the floor.

Specifically the evaluators assessed the following components and an average score was obtained for each item:

Communication and coordination mechanism (Scenario 1 and 2) ***Maximum score =12***
  - Roles and responsibilities of different stakeholders in case of outbreak
  - Clarity on communication protocol
  - Effectiveness of coordination mechanism
Initial outbreak response (Scenario 3 and 4) ***Maximum score = 20***
  - Ability to develop initial response plan
  - Ability to plan for enhancing surveillance
  - Ability to plan for vaccination response within 14 days
  - Ability to plan for effective Soc. Mob activities for first vaccination responseo
  - Ability of program for responding to media
Quality of plans and documents ***Maximum score = 16***
  - Quality of 6 month response plan - Cross checked if it was comprehensive and therefore including all components.
  - Quality of Situational Report (SITREP) - Cross checked if it had all relevant components.
  - Media briefing points - Cross checked if it was appropriate.
  - Quality of surveillance improvement plan
Flexibility (scenario 5, 6 and 7) ***Maximum score = 8***
  - Ability to modify response as per new information
  - Capacity to respond to new challenges

The overall ability of the country to respond to outbreak was summed up from all the above analysis. Each of the above components was assessed accordingly on a 5-Likert scale and graded as shown below;

0 = No response (where expected activity was not carried out)
1= Poor (where activities were unsuccessfully conducted)
2 = Average (where performance was reasonable)
3= Good,
4= Very good

The scores are converted to percentages by diving the actual with the expected maximum score and multiplied by hundred.

## Results

### Review of national plans

All seven countries had approved or draft national polio plans available, which were considered critical for successfully responding to any poliovirus outbreak. They all emphasized the need for comprehensive national plans in each exercise. The plans varied in degree of detail, review date, and connectivity to generic disease outbreak plans. National plans were too general and failed to specify all details important for their implementation. Some of the strength of the plans include changes in the plan were done and relevant as well as the communication part and provisions were made for social mapping for mobile population for the micro planning. The plans had target age group well defined for the first and second rounds of vaccination and dates and plans were well explained. Teams modified the plan after results from the extra information and key planning and SIAs component were well addressed. Specific strategies for security constraints in the areas were explained roles of other partners / NGOs were clear in the plan and actively participated in team work. Plans for Independent Monitors and Lot Quality Assurance (LQAs) to validate campaign results were clearly stated. Furthermore, Group seemed to know what they needed for the SITREP. Presented SITREP complete and organized using the standard template with detailed information. The SITREP covered previous trends of vaccination and polio outbreaks and collaboration between groups was observed.

Some of the major weaknesses identified in national plans included planning assumptions based on unreliable routine immunization coverage data, weak communications components, and lack of clarity on national vaccine policies and sources of procurement in case of an outbreak. Combined plan was available based on expertise and experiences but not in line with the presented polio outbreak preparedness and response plan. Challenges mentioned in the SITREP but no mitigation plans explained and stakeholders were not given copies of SITREP.

### Communication and coordination mechanism

The different stakeholders involved in the polio outbreak response activities were present. The communication activities were efficiently discharged with relevant stakeholders adequately informed. Verbal communication from between relevant levels of communication was efficiently conducted; it was immediate and moved smoothly initially. The communication used the standard protocol within the country and also neighboring countries (IHR). Communication was clear and moved down the line and content of information shared by phone (specifying VDPV2). National social mobilization committee (NSC) was active and provides directions to make things move forward. Sub-committees began to work with clear guidance and follow up from the NSC and how the sub committees would coordinate amongst each other. The Subcommittees reported on additional requirements to NSC and Emergency operations center (EOC) was established immediately.

However, a number of gaps were observed, some of which included the fact that reference to the response plan was not done whenever was necessary to be clear what needs to be done at each level. The plan actually was shared in scenario 1 in many of the countries involved. There was a delay in making decision on what needs to be done immediately and initial confusion to the National Steering Committee was noticed because of lack clarity of information. Roles and responsibility of each partner was not clear and internal communication in the agencies was not evident. Furthermore, formal communication between stakeholders was not evident in many of the countries. The national preparedness and response plan did not show clearly committees TORs and responsibilities and no evidence of outbreak to be declared as public health emergency of international concern (PHEIC) by Minister rather than reported information in some of the countries. Worse still, in many of these countries,WHO and UNICEF Representatives did not physically participate yet they are key to effective outbreak response.

### Initial outbreak response

With respect to ability to develop initial response plan, governments in all the countries were clearly committed in the process and took leadership role in all teams. All the teams were able to develop initial plans, where communication committee planted people in different committees so that they were well informed about what the other groups were doing. The plans were presented after the sessions with clear timelines and responsible persons for all sub- committee. Clear risk assessment criteria were used for initial outbreak response and the plans and budgets prepared for various subcommittees were clear. Furthermore, clear basic information on the decision on the date of the first response and target population was uniform and clear to all teams.

With respect to ability to plan for enhancing surveillance, the countries managed to develop, present and implement surveillance enhancement plan with time line. There was active participation of MOH and partners and other committees in the detail case and social investigation. The planned procedures and investigation details were in line with revised SOPs. Investigation teams were used to conduct cold chain assessment and evaluate security status of the area. The availability of campaign operation plan (logistics, communication, training and monitoring plan) with central team deployment and time line and pre, intra and post campaign monitoring were in place. Availability of mOPV request form was filled and initial plans were developed with budget. Communication plan was in place. Furthermore, the countries ensured that distribution plans of vaccines were developed with clear communication and links with all subcommittees and subnational level to ensure that a comprehensive plan for the country is developed. Communication teams were very effective in the different countries. They broke into sub groups and delegated responsibilities and came up with a clear response plan. Active inter committee information sharing with clear campaign dates, age group and communication for development strategy. MOH lead role with active participation of partners was clearly noticed in all the countries. Media kit was developed and rumors logged handled by Director of Health Promotion and well designed and coordinated media talking notes developed. There was good collaboration between partners in responding to rumors.

However, some areas of weakness were observed. These include the absence of clear guide in the national polio outbreak preparedness and response plan. Other counties to be involved in the initial response were not adequately involved in the outbreak response. No outbreak investigation reporting format and checklist was developed or shared and there was no timeline of activities presented in the Gantt chart developed in some countries. Furthermore, the scope of the response was not well defined and clear to all committees and message for caretakers vaccination mixed house to house and to health facilities lacked clarity.

### Flexibility

Communication teams in the different countries were more effective and knew what they needed to do. This was very evident in South Sudan. The teams had better understanding of their roles and developed better ability to modify the response. Strong field experience was used to address the scenario and consultation with other teams became visible in modifying plans for communication, operations and surveillance. Vaccine distribution plan was feasible and committees initially took a short time to organize themselves and respond quickly on the scenarios. At the end of the session, most teams were able to modify their plans to reflect the new information. All committee demonstrated high degree of flexibility to respond to new challenges including cross border activities. Special groups were covered and consideration of review of entire country risks. Consideration of wide and detailed coordination, collaboration and engagement of all important stakeholders among community mobilizers were clearly demonstrated. The technical ability and capacity to respond to any polio event or outbreak is available. The teams prepared fast track plan for funds disbursement from National level to states level (shared budget for accountability and transparency to all key stakeholders) and ensured rapid clearance for funds transfer.

However, limited direct involvement of UNHCR in the planning process was noticed, Communications were expected through NGOs working closely with UNHCR but this did not come out clearly n the plans. The inter country synchronization of effort was limited and there was delayed HR assessment to know the required capacities. Following information of the new case, EOC delayed to guide the various committees.

### Overall ability of country to respond to outbreak

The technical ability and capacity to respond to any polio event or outbreak were demonstrated in the countries. There were prepared fast track plans for funds disbursement from National to district level (shared budget for accountability and transparency to all key stakeholders) and ensured rapid clearance for funds transfer though with limited reflection of practical experience to the National Polio Outbreak Preparedness and Response plan. Remarkable participants dropout rates in day two compared to day 1 and limited participation of UNHCR was observed.

## Participants Evaluation

The participants’ evaluation closely mirrored the findings and comments of the evaluation team. This basically strengthens the need for the countries to work on the POSE findings and recommendations on the need to review the national response plan. All participants agreed that, the content and format of the Polio Outbreak Simulation Exercise in each country met the objective. The following is the summary of key observations reported from the participant’s evaluation;

- Command Post direction is important in implementation of the exercise
- It is good to have preparedness meeting involving all partners with the Command Post
- There were discrepancies between the national outbreak response plan and SOPs
- Inadequate time for completing the exercise effectively, need at least three days
- Documents for the exercise; National plan and SOPs not shared on time.
- Awareness was created on how to respond to polio emergency
- National Outbreak Response Plan needs to be revised as current plan lacks details and functionality of key groups.
- The exercise generated important issues and identified useful lessons and also it identified several gaps in the national preparedness plan
- Scenarios should involve field visits

## Discussion

Generally, the National Polio Response Plan is in line with the revised polio outbreak SOPs. However most of response details are missing including the committees, sub-committees and tasks force membership and detailed functions. The country team of South Sudan composed of well experienced professional staff that actually simulated according to the SOPs. The exercise stimulated participants and decision makers in updating the missing information in the National polio outbreak preparedness and response plan.

On the whole the external evaluators observed that the technical capability to respond to an outbreak of polio was available in the different countries with some few gaps which can be addressed while performing future POSE. The insecurity challenge resulted in non-participation of State Ministry of Health participants and therefore a consideration to cover them in regard to the fact that, some area where there is more likelihood of experiencing outbreak. The external facilitators work was appreciated by participants. The Ministries of Health recognized the importance of POSE and its applicability to any disease outbreak and requested further support in future simulation exercises. The commitment of staff was well appreciated with evaluators and facilitators alike.

The impact of simulation exercises to date, along with positive feedback from the participants, suggested that the POSEs have become valuable tools that are helping to respond to polio outbreaks. The exercises helped familiarize participating countries with each other’s preparedness plans and practices and promoted better understanding and cooperation between countries and international organizations^2,3-5^. They fostered discussions, proposed realistic actions, and identified important issues and areas for development^2^. The experiences and lessons learned from these exercises are transferable to other vaccine-preventable disease

The following is the list of recommendations which are expected to be implemented by the Ministry of Health in collaboration with key immunisation partners.

- The Ministry of Health in collaboration with key immunisation partners should revise the National polio outbreak preparedness and response plan and reorganize the relevant national-level decision making bodies or committees that may exist should an outbreak occur and align to SOPs by 31^st^ December, 2017.
- Early engagement of all partners important stakeholders dealing with security and refuges e.g. UNHCR, Security agencies, Immigration Department etc as part of the team in the revision process of the National polio outbreak preparedness and response plan and follow up POSE or actual outbreak event.
- The Ministry of Health should consider conducting a similar POSE to include participants from the that were not included as soon as possible.

## Conclusion

Polio outbreak simulation exercises can be valuable tools to help maintain polio-free status and should be extended to other high-risk countries and subnational areas in the HoA Region and elsewhere. However, there is need to standardize the process and methods for conducting POSE for comparability. In this analysis, we could not make use of the rich quantitative data presented in some countries like Uganda, because most of the other countries did not go through the same rigour and quantification of the outcomes of the exercise, thus making comparability impossible. As the programme moves to implement this in other countries it is important to standardize the method across the region and ensure the use of uniform template for reporting results.

## Figures and Tables

**Table 1:** Evaluation sheet: Polio Outbreak Simulation exercise Evaluator is expected to observe the simulation exercise and evaluate the response of group to the scenario. Evaluator is also expected to provide specific area of concerns where the response could be better. Score: 0=No response, 1= Poor, 2= Average, 3= Good, 4= Very good

	Component	Score	Comments
	**Communication and coordination mechanism (Scenario 1 &2)**		
1	Roles and responsibilities of different stakeholders in case of outbreak	0/1/2/3/4	
2	Clarity on communication protocol	0/1/2/3/4	
3	Effectiveness of coordination mechanism	0/1/2/3/4	
	**Initial outbreak response (Scenario 3 & 4)**		
4	Ability to develop initial response plan	0/1/2/3/4	
5	Ability to plan for enhancing surveillance	0/1/2/3/4	
6	Ability to plan for vaccination response within 14 days	0/1/2/3/4	
7	Ability to plan for effective Soc. Mob activities for first vaccination response	0/1/2/3/4	
8	Ability of program for responding to media	0/1/2/3/4	
	**Quality of plans and documents**		
9	Quality of 6-month response plan (Is it comprehensive-includes all components?)	0/1/2/3/4	
10	Quality of SITREP (Is it having all relevant component?)	0/1/2/3/4	
11	Media briefing points (Is it appropriate?)	0/1/2/3/4	
12	Quality of surveillance improvement plan	0/1/2/3/4	
	**Flexibility (scenario 5 & 6)**		
13	Ability to modify response as per new information	0/1/2/3/4	
14	Capacity to respond to new challenges	0/1/2/3/4	
15	Overall ability of country to respond to outbreak	0/1/2/3/4	

**Table 2:** Characteristics of Polio Outbreak Simulation Exercises (POSEs) in Horn of Africa (HoA) Held between 2016 and 2017

Characteristics	POSE 1	POSE 2	POSE 3	POSE 4	POSE 5	POSE 6	POSE 7
Date and location	26-27 January 2016 Sudan	22-23 June, 2016; Tanzania	18-22 July 2016; Asma- ra, Eritrea	12-19 October, 2016; Ethiopia	27 - 29 March 2017; Kenya	7-8 November 2017, South Sudan	29-30 November, 2017; Uganda
Number of persons	84 (Including exercise support personnel and facilitators	90 (Including exercise support personnel and facilitators	59 (Including exercise support personnel and facilitators	49 (Including exercise support personnel and facilitators	58 (Including exercise support personnel and facilitators	45 (Including exercise support personnel and facilitators	42 (Including exercise support personnel and facilitators
Participants	Federal Epi staff; States Operational Managers; WHO National Medical Officers; Central Cold Chain Staff; WHO Staff, UNICEF staff; Laboratory focal persons, EOC staff, RRT, NTF members; Representatives from the locality and Concerned Organizations/ agencies	MoH, DG Health, Permanent Secretary and other top MOH Officials; MOH/DEG; TRCS; MITI; WAMJW; PORG; IVD Staff; WHO; UNICEF; CHAI; USAID/ MCSP; PATH; NTF; District representatives	MoH of Health staff of EPI, IDSR; Health Promotion; Data Managers; Director of Hospital at National; Zoba and Hospital levels; WHO; UNICEF; Religious leaders;	MoH Ethiopia; WHO; UNICEF; CORE Group; USAID; CCRA/ CORE; Rotary; Represen- trative of various regions and Federal Government Organization	MoH, WHO, UNICEF, Kenya Red Cross, KEM- RI, CDC, CORD, CHAI,	BMGF, WHO, MSF. ADA, CCC, Medcair, UNICEF, IOM, CORE group, Livewell, WHO-HOA, ACF, ARUDA, UNICEF, UNIDO, MOH etc	MoH, WHO Uganda, WHO Nigeria, WHO HoA, UNICEF Uganda, UNICEF Esaro,
Observers	GPEI Horn of Africa Coordination Office; WHO/ AFRO; UNICEF ESARO	GPEI Horn of Africa Coordination Office; WHO/AFRO; UNICEF ESARO	GPEI Horn of Africa Coordination Office; WHO/ AFRO; UNICEF ESARO	GPEI Horn of Africa Coordination Office; WHO/AFRO; UNICEF ESARO	GPEI Horn of Africa Coordination Office; WHO/AFRO; UNICEF ESARO	GPEI Horn of Africa Coordination Office; WHO/AFRO; UNICEF ESARO, IST/ ESA,	GPEI Horn of Africa Coordination Office; WHO/ AFRO; UNICEF ESARO, IST/ESA, WHO/Nigeria
Sample Scen- erios	Scenario 1 On 13th August, UVRI Polio lab gives a call to UNEPI Manager that EPID No. UGA-KAA- KAA-15-008 is positive for WPV1. This case is from Kaabong district with date of onset 15-07-2015. (UVRI Polio Lab to call UN- EPI Manager) Exercise (20+5 min): Conduct the communication and coordination activities as required immediately after notification of WPV (within 6 hrs). List the additional information to be collected for deciding initial response plan	Scenario 1 On 13th August, UVRI Polio lab gives a call to UNEPI Manager that EPID No. UGA- KAA-KAA-15-008 is positive for WPV1. This case is from Kaabong district with date of onset 15-07-2015. (UVRI Polio Lab to call UNEPI Manager) Exercise (20+5 min): Conduct the communication and coordination activities as required immediately after notification of WPV (within 6 hrs). List the additional information to be collected for deciding initial response plan	Scenario 1 On 13th August, UVRI Polio lab gives a call to UN- EPI Manager that EPID No. UGA-KAA- KAA-15-008 is positive for WPV1. This case is from Kaabong district with date of onset 15-07-2015. (UVRI Polio Lab to call UN- EPI Manager) Exercise (20+5 min): Conduct the communication and coordination activities as required immediately after notification of WPV (within 6 hrs). List the additional information to be collected for deciding initial response plan	Scenario 1 On 13th August, UVRI Polio lab gives a call to UNEPI Manager that EPID No. UGA-KAA- KAA-15-008 is positive for WPV1. This case is from Kaabong district with date of onset 15-07-2015. (UVRI Polio Lab to call UNEPI Manager) Exercise (20+5 min): Conduct the communication and coordination activities as required immediately after notification of WPV (within 6 hrs). List the additional information to be collected for deciding initial response plan	Scenario 1 On 13th August, UVRI Polio lab gives a call to UNEPI Manager that EPID No. UGA-KAA- KAA-15-008 is positive for WPV1. This case is from Kaabong district with date of onset 15-07-2015. (UVRI Polio Lab to call UNEPI Manager) Exercise (20+5 min): Conduct the communication and coordination activities as required immediately after notification of WPV (within 6 hrs). List the additional information to be collected for deciding initial response plan	Scenario 1 On 13th August, UVRI Polio lab gives a call to UNEPI Manager that EPID No. UGA-KAA- KAA-15-008 is positive for WPV1. This case is from Kaabong district with date of onset 15-07-2015. (UVRI Polio Lab to call UNEPI Manager) Exercise (20+5 min): Conduct the communication and coordination activities as required immediately after notification of WPV (within 6 hrs). List the additional information to be collected for deciding initial response plan	Scenario 1 On 13th August, UVRI Polio lab gives a call to UN- EPI Manager that EPID No. UGA- KAA-KAA-15-008 is positive for WPV1. This case is from Kaabong district with date of onset 15-072015. Conduct the communication and coordination activities as required immediately after notification of WPV (within 6 hrs). List the additional information to be collected for deciding initial response plan
Additional background information	Although wild poliovirus transmission was interrupted, Sudan continues to recognize the risk of importing wild poliovirus as long as it is still circulating anywhere else in the world. The country had actually faced four importations during 2004, 2007, 2008 and 2009.	Tanzania is located within the HoA region in Africa, and has boundaries with Kenya, Uganda, South Sudan and Ethiopia, all threatened with poliovirus outbreaks. It is home to two renowned tourism destinations, which attracts huge populations of tourists every year, coupled with its 55.5 million population, an outbreak of poliovirus came become a major global public health challenge.	Eritrea is located in the Horn of Africa, with a population of approximately 3,599,384 in 2016. Eighty percent 80% of the people lived in rural and semi urban areas. The country has 58Sub Zobas 715 administrative areas and 2,564 villages.	Ethiopia is located in the north eastern part of Africa, also known as the Horn of Africa, lies between 3 and 15 degrees north latitude and 33 and 48 degrees east longitude. It has the tenth largest land mass in Africa, covering 1,104,300 square kilometers.	Kenya is located in the HoA region and it bordered with Tanzania, Uganda, Ethiopia, South Sudan and Somalia. Kenya has a projected total population for 2017 of 47,735,775 of which 1,548,011 (3.2%) are infants, 7,507,409 (15.7%) children under 5 years, and 18,670,226 (39.1%) under – fifteen 15 years.	South Sudan is located within the HoA region, which experienced outbreak of polio virus in 2013. The projected population for 2017 using 3.0% growth rate from the census figures for 2008 is 12,979,639. The last indigenous case of wild poliovirus in South Sudan was reported in 2001. Since then South Sudan has experienced wild polio outbreaks in 2004/2005and 2008/2009 as a results of importation.	Uganda is a landlocked country located in Eastern Africa bordering South Sudan to the North, Kenya to the East, Tanzania and Rwanda to the South, and DRC to the West covering approximately 241,039 square kilometers of which 18%pres- ent Lake Victoria and other lakes with an estimated population of 37 Million projected for 2017 using 3.0% growth rate from the 24.2 Million figure for 2002.

## Box 1 Polio Outbreak response table top simulation exercise

**1.**   Simulation scenarios

**3.1**   Scenario 1

On 13^th^ August, UVRI Polio lab gives a call to UNEPI Manager that EPID No. UGA-KAA-KAA-15-008 is positive for WPV1. This case is from Kaabong district with date of onset 15-07-2015. (*UVRI Polio Lab to call UNEPI Manager*)

**1.0.1  Exercise (20+5 min):**

- Conduct the communication and coordination activities as required immediately after notification of WPV (within 6 hrs).
- List the additional information to be collected for deciding initial response plan

**3.2**   Scenario 2

A meeting of NTF is called by Office of DG Health in 6 hrs, all of you are part of it.

***UNEPI** and WHO present following information about the case and area:*

- CIF shows that the EPID No. UGA-KAA-KAA-15-008 is of a child name Samuel Sabuni Lochuk, age 6 years. Child has received 6 doses of OPV (2 RI and 3 SIA doses).
- The district has hard to reach areas (case is from Timu, hard to reach part of district)
- Kabong district has NPAFP rate of 0
- Penta coverage of area is 74% in 2014.
- The district has conducted 2 SIAs in 2013, none in 2014 and 1 in Feb 2015.

**1.0.2  Exercise (40+10 min):**

- NTF to meet and take the key decisions and actions.

*Specific outcomes from the meeting of NTF:*

- Do the risk assessment (use the available High risk areas table) and develop initial response plan.
- Decide on actions related to IHR recommendations
- Activate subcommittees, RRT and EOC
- Put the coordination mechanism in place
- Send the communications as appropriate

**3.3  Scenario 3**

NTF activates outbreak response plan, subcommittees, RRT and EOC.

NTF has decided following:

- Detail epidemiological-social investigation of case should be conducted at the earliest.
- First vaccination response should be conducted with bOPV on 27^th^ August covering 5 districts around Kaabong (Kitgum, Kaabong, Moroto, Kotido, Napak and Abim).
- Enhance surveillance activities should be implemented.
- Media strategy will be reactive rather than proactive
- NTF declared outbreak as public health emergency.

**1.0.3  Exercise (40+10 min):**

- Conduct the activities to be done within 24 and 72 hrs

*Specific outcomes*

Surveillance:

- List/ take the actions for detail investigation of outbreak
- Develop surveillance enhancement plan with timelines, activities and person responsible
- Develop and send communications to concerned persons on surveillance enhancement activities.

Vaccination response:

- Assess the vaccine and funds required for campaign and communicate to GPEI (through WHO & UNICEF)
- Develop a plan for conducting vaccination response with timelines, activities and person responsible and communicate to the districts involved.
- Develop cold chain and logistics need assessment, procurement and distribution plan.
- Develop plan of action for implementing IHR recommendations and communicate to concerned officials.

Media and Soc. Mob:

- Develop Soc. Mob and communication plan including key C4D messages for initial vaccination response.
- DG Health has called you for a meeting on how to handle media. Prepare a summary of media strategy to discuss with her.

**3.4  Scenario 4**

Its 16^th^ August.

There has been news in TV on the case and *DG Health, UNICEF Rep and WR have received multiple media queries.* In view of this, DG health has called a press briefing and invited WR and UNICEF Rep also to participate in that.

*WHO RO and GPEI’s OPRTT Chair have called up to Polio control room to get the update and ask for additional support needed.*

*Report on detailed investigation of outbreak received* showed that the particular community to which child belongs is Nomadic community and moves frequently across the border to South Sudan and Kenya. During the active case search team found evidence of missed AFP cases, one of which is hot case. Although not hostile, there is not much demand for vaccination.

**1.0.4  Exercise (40+10 min):**

- Develop SITREP and share with all stakeholders including global partners including the extra support required
- Develop talking points for media briefing and brief DG and other spokespersons
- Modify Soc. Mob plan in light of this new information.
- Develop 6 month outbreak response plan and share.
- If needed, modify vaccination response plan in the light of new information from outbreak investigation and communicate.
- Take urgent actions to improve surveillance in the area

**3.5  Scenario 5**

WHO IST has called and asked for a teleconference with following participants:

- WHO HQ, WHO IST, UNICEF ESARO and CDC
- Country team:
  - MoH, UVRI Lab, EOC (IMT)
  - WHO
  - UNICEF
  - CDC

Agenda for meeting:

- Situation update
- Response plan
- Support needed from GPEI
  - Human resources
  - Funds
  - Vaccine
  - Other

**1.0.5  Exercise**

Do the role play

**3.6**  Scenario 6

Its 21^st^ August. GPEI has sent 3 support (Technical, Operations and communication) and they are in country.

Funds have not been disbursed to the field yet from national level.

*There is a call from UNICEF SD that vaccine will be reaching to country on 24^th^ August.*

*Polio Control Room (EOC) received the information from GPEI RRT* that preparations for vaccination campaign at the district and county level in Kaabong is suboptimal. Field level microplanning has not started, there is no plan to focus on coverage of nomadic population and training of vaccinators is yet to be rolled out.

**1.0.6  Exercise (40+10 min):**

- Take actions to fast track the fund disbursement process
- Modify vaccine distribution plan to ensure vaccine reached by 25^th^ to district level.
- List the actions you will take to ensure better preparation and good quality activity in the district Kaabong.
- Take actions to integrate SM activities in overall preparatory activities.

**3.7  Scenario 7**

Its last day of first vaccination response and *Polio Control Room (EOC) received the information* that 4 villages in Kaabong have refused vaccination due to religious reasons and two villages were missed from vaccination.

*PCR also received the information* that nomadic population have not been covered well.

*UVRI Polio Lab informs about one new case notified from Kaanuagu district in south of Uganda*

**1.0.7  Exercise (30+10 min):**

- List the specific actions you will take in response to this feedback on activity.

Modify your outbreak response plan in light of this new information.
